# Non-invasive algorithm for bowel motility estimation using a back-propagation neural network model of bowel sounds

**DOI:** 10.1186/1475-925X-10-69

**Published:** 2011-08-10

**Authors:** Keo-Sik Kim, Jeong-Hwan Seo, Chul-Gyu Song

**Affiliations:** 1 School of Electronics and Information Engineering, Chonbuk National University, 664-14 1 Ga, Deokjin-dong, Jeonju, Republic of Korea; 2 School of Medicine, Department of Rehabilitation Medicine, Chonbuk National University Hospital, 634-18 Geumam-dong, Jeonju, Republic of Korea; 3 Center for Advanced Image and Information Technology, Chonbuk National University, 664-14 1 Ga, Deokjin-dong, Jeonju, Republic of Korea

## Abstract

**Background:**

Radiological scoring methods such as colon transit time (CTT) have been widely used for the assessment of bowel motility. However, these radiograph-based methods need cumbersome radiological instruments and their frequent exposure to radiation. Therefore, a non-invasive estimation algorithm of bowel motility, based on a back-propagation neural network (BPNN) model of bowel sounds (BS) obtained by an auscultation, was devised.

**Methods:**

Twelve healthy males (age: 24.8 ± 2.7 years) and 6 patients with spinal cord injury (6 males, age: 55.3 ± 7.1 years) were examined. BS signals generated during the digestive process were recorded from 3 colonic segments (ascending, descending and sigmoid colon), and then, the acoustical features (jitter and shimmer) of the individual BS segment were obtained. Only 6 features (*J_1, 3_, J_3, 3_, S_1, 2_, S_2, 1_, S_2, 2_, S_3, 2_*), which are highly correlated to the CTTs measured by the conventional method, were used as the features of the input vector for the BPNN.

**Results:**

As a results, both the jitters and shimmers of the normal subjects were relatively higher than those of the patients, whereas the CTTs of the normal subjects were relatively lower than those of the patients (*p *< 0.01). Also, through *k*-fold cross validation, the correlation coefficient and mean average error between the CTTs measured by a conventional radiograph and the values estimated by our algorithm were 0.89 and 10.6 hours, respectively.

**Conclusions:**

The jitter and shimmer of the BS signals generated during the peristalsis could be clinically useful for the discriminative parameters of bowel motility. Also, the devised algorithm showed good potential for the continuous monitoring and estimation of bowel motility, instead of conventional radiography, and thus, it could be used as a complementary tool for the non-invasive measurement of bowel motility.

## Background

Radiological scoring methods such as the Barr and Blethyn scores [1] and colon transit time (CTT) [2,3], which operate by means of X-rays and magnetic resonance imaging (MRI), have generally been used for the assessment of bowel motility. Among these methods, the CTT described by Metcalf [2] is considered to be the gold-standard. It is simply assessed by measuring the movement of radiopaque markers taken at a fixed time after their ingestion. This test is highly reproducible and most useful in determining whether constipation symptoms are associated with normal or slow transit. However, these radiograph-based methods need an expensive, cumbersome radiological instrument and their frequent exposure to radiation.

In an effort to resolve these limitations, assessing bowel motility using bowel sound (BS) signals obtained by means of auscultation has been recently attempted. Tomomasa *et al. *[4] and Craine *et al. *[5,6] presented changes in various features (sound-/motility-index, sound-to-sound interval, number of events and so on) of bowel sound according to the pathological condition. Yamaguchi *et al. *[7] showed that the sound index of the gastro-duodenal sound in the diabetes mellitus patients was significantly lower after food intake than in healthy adults. Also, wavelet-based strategies for the signal acquisition, de-noising, automated segmentation, event detection and feature extraction of bowel sounds were proposed [8-11]. Dimoulas *et al. *[12] implemented an autonomous BS monitoring system utilizing wavelet feature extraction and multi-layer perceptrons (MLP) network classifiers for the pattern classification of BS segments. Besides, the fractal-dimension analysis of BS signals [9,10,13], principal component analysis (PCA) [14], Weiner filtering [15] and hybrid expert system using hierarchical audio pattern recognition [16] have been tried to detect the informative feature of BS and evaluate the bowel motility via an auscultation. These BSs are generated from the movement of the intestinal contents and gas in the lumen of the gastrointestinal tract during peristalsis; therefore, they can be used clinically as useful indicators of bowel motility.

Therefore, the aim of this study is to develop a non-invasive estimation algorithm of bowel motility, based on an artificial neural network (ANN) model of the jitter and shimmer, which were considered as useful features in recent study [17], of the BS signals during the digestive process. Also, we derived an ANN model between the acoustical features obtained from the BS signals and measured CTT, and finally determined the feasibility of the proposed method.

## Methods

Twelve healthy men (age: 24.8 ± 2.7 years, BMI: 23.6 ± 2.7 kg/m^2^), not taking any medication that might affect their bowel motility, and 6 patients (6 men, age: 55.3 ± 7.1 years, BMI: 24.0 ± 3.5 kg/m^2^) with delayed gastric emptying due to spinal cord injury were examined. The average number of months after injury was 17.2 ± 28.5 months. Ethical approval for this study was obtained from the Institutional Review Board (IRB) of Chonbuk National University Hospital.

The total CTTs were measured by the strategy described by Metcalf et al. [2]. The subject ingested a radiopaque marker capsule containing 20 markers (Kolomark™, Korea) each day for 3 days. On days 4 and 7, an abdominal X-ray image was obtained. Also, on day 7, the 3-channel BS signals were obtained from the right upper (ascending colon, CH1), left upper (descending colon, CH2) and left lower quadrants (sigmoid colon, CH3) of the abdomen with the subjects lying on a bed, respectively. Under fasting conditions, the subjects took test meals of 200 g at 9:00 AM. After that, the data was recorded for 10 minutes at 9:30 AM (Post1), 1:00 PM (Post4) and 5:00 PM (Post8). The subjects were asked to take no food from 9:30 AM to 5:00 PM.

**Figure 1:**
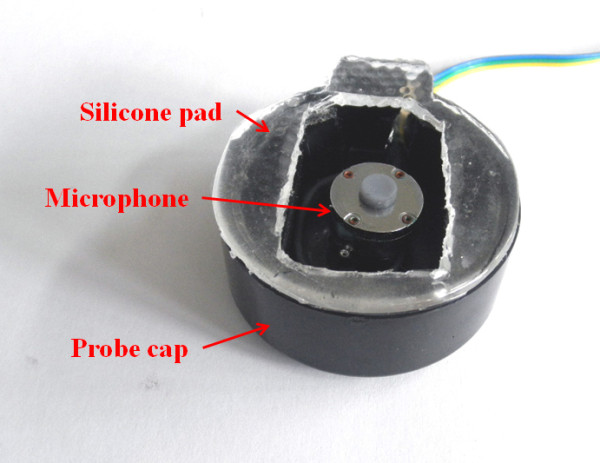
**Sensor-adaptation set-up of noncontact-type probe**.

A piezo-polymer microphone (CM-01B, Measurement Specialties Inc., U.S.) with a frequency bandwidth of 8-2, 200 Hz was used for collecting the BS signal. The frequency content of the BS signals is known to be energetic mainly between 100 and 500 Hz [11,18], thus the collected BS signals were pre-processed through a 60 Hz notch filter for removing the power noise and 5-600 Hz band-pass filter for reducing the motion artefact noise caused by respiration activation and unwanted noise. After that, the signals were digitized by an A/D converter (USB-6009, National Instruments™, U.S.) at a sampling rate of 8 KHz and resolution of 14 bits. Figure 1 shows the sensor-adaptation set-up of the noncontact-type probe used for recording BS signals.

The statistical analysis was conducted using SPSS (ver. 14, SPSS Inc., U.S.) software. Unpaired T-test was performed to compare the features obtained from the normal subjects with those from the patients. Also, Pearson's correlation coefficients were obtained to evaluate the relationship between the acoustical features by means of our algorithm and CTT by means of the conventional method. The level of statistical significance was set as *p *< 0.05. Finally, *k*-fold cross-validation was performed to evaluate the performance of our algorithm.

## Bowel motility estimation

### BS detection and segmentation

**Figure 2:**
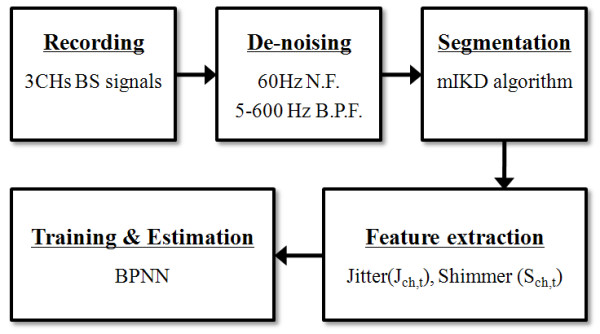
**Procedure used for the estimation of the colon transit time (CTT) using the jitter and shimmer of bowel sounds**.

Figure 2 shows the procedure used for detecting selectively the BS segments from noisy BS signals and extracting the features, in order to estimate the CTT using the ANN model. First, the recorded BS signals were processed using the modified iterative kurtosis-based detection (mIKD) algorithm, devised in our previous study [17], for the selective detection of BS segments through noise-gating. Significant deviations from kurtosis value can be attributed to the presence of non-Gaussian signals such as the BS, since kurtosis is theoretically zero for Gaussian signals such as back-ground sound (BGSs) signals [19]. Next, the detected BS signals were divided into the individual BS segments, where an individual BS segment is defined as sounds having duration larger than 20 msec without the period of silence [11].

### Feature extraction

The characteristic parameters, viz. the absolute jitter (*J_ch, t_*) and shimmer (*S_ch, t_*), of each BS segment, were calculated using (1) and (2), respectively;(1)(2)

where *P_i_*, *A_i _*and *N *are the peak-to-peak period and amplitude of each pitch and the number of pitches, while *ch *and *t *are the channel number (1 = CH1, 2 = CH2 and 3 = CH3) and time index (1 = Post1, 2 = Post4 and 3 = Post8) when the BSs were recorded, respectively. The jitter is the average absolute difference between consecutive periods, while the shimmer is the average absolute difference between the amplitudes of consecutive periods in decibels [20].

A total of 18 features (the *J_ch, t _*of 9 and *S_ch, t _*of 9) per subject were obtained. Among all of these 18 features, the features highly correlated with the measured CTT were selected as the informative one through correlation analysis. As a result, only the top six features (*J_1, 3_, S_1, 2_, S_3, 2_, J_3, 3_, S_2, 2_, S_2, 1_*) with the high correlation coefficient of 0.65 and over were used as the features of the input vector for the ANN. Table 1 represents the correlation coefficients between the selected features and CTT and their *p*-values.

**Table 1:** Correlation coefficients (C.C.) between the selected features and measured colon transit time (CTT) and their *p*-values

	J_1, 3_	S_1, 2_	S_3, 2_	J_3.3_	S_2, 2_	S_2, 1_	CTT
**N1**	0.154	37.6	36.1	0.159	35.1	35.4	10.8
**N2**	0.172	34.4	34.2	0.167	35.4	35.6	26.4
**N3**	0.176	38.3	36.6	0.177	38.2	37.0	10.8
**N4**	0.173	34.7	32.4	0.166	35.1	33.8	2.4
**N5**	0.162	34.6	36.8	0.155	36.3	38.3	18.0
**N6**	0.164	34.6	34.1	0.168	35.2	35.0	12.0
**N7**	0.160	37.1	36.5	0.164	34.7	35.2	16.8
**N8**	0.161	34.5	35.0	0.164	35.7	37.5	60.0
**N9**	0.157	36.2	33.8	0.168	35.4	37.3	26.4
**N10**	0.159	36.0	36.0	0.161	34.6	38.2	25.2
**N11**	0.167	35.0	36.7	0.165	34.3	36.4	2.4
**N12**	0.163	32.3	32.3	0.161	35.8	33.1	33.0
**P1**	0.132	30.2	31.1	0.151	30.1	29.8	69.0
**P2**	0.139	32.0	27.8	0.148	32.6	31.9	86.0
**P3**	0.147	31.8	30.6	0.154	31.7	32.4	102.0
**P4**	0.149	30.9	34.5	0.156	35.0	31.9	66.0
**P5**	0.134	32.5	30.8	0.142	30.7	30.5	82.2
**P6**	0.155	29.9	30.9	0.155	30.5	30.7	68.0

**C.C**.	-0.79*	-0.76*	-0.75*	-0.74*	-0.72*	-0.68*	

N: normal subject, P: patient, J_ch, t_: jitter, S_ch, t_: shimmer, *: *p *< 0.01

### Architecture of the ANN

The estimation of the CTT was performed using a back-propagation neural network (BPNN). The input and output layers of the BPNN consisted of 7 nodes (selected 6 features and 1 bias) and 1 node (estimated CTT, eCTT), respectively. The training of a network by back-propagation involves three stages; the feed forward of the input training pattern, the calculation and back-propagation of the associated error, and the adjustment of the weights. After training, the application of the net involves only the computations of the feed-forward phase [21].

Also, the performance of a BPNN can depend on its structure such as the learning rate and number of hidden nodes. Thus, we determined their practical values which can provide the best performance, i.e. the least error and best correlation between the measured CTT and estimated one. The values of learning rate tested were 0.05, 0.1, 0.2, 0.3 and 0.4 (5 cases), and the values of the number of hidden nodes were 2, 3, 4 and 5 (4 cases).

## Results

**Figure 3:**
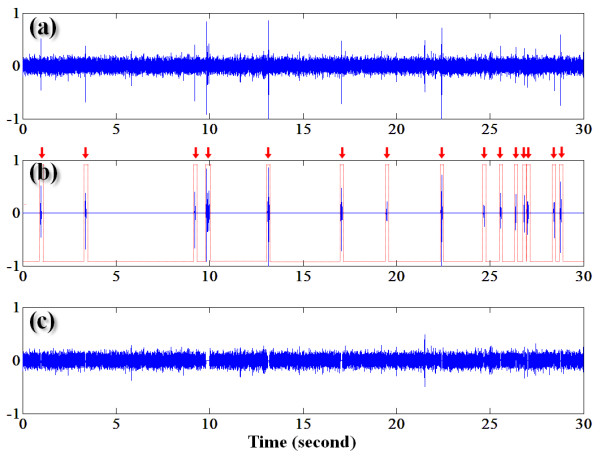
**a) Raw bowel sound signals obtained from the abdomen**. b) Inherent bowel sounds detected by the modified iterative kurtosis-based detector algorithm. c) Background noise. Arrows represent the individual bowel sound segments.

Figure 3 shows (a) the raw signals obtained from healthy subject (31-year-old male, ascending colon at Post1), (b) BS segments detected by the mIKD algorithm and (c) background noise. As shown in the output of the mIKD (in Figure 3b), our algorithm could sensitively separate even the low peaks (e.g. around time 19.5 seconds) although they were hard to be distinguished from BGSs by means of visual or auditory inspection. On the contrary, the high peak (e.g. around 21.5 seconds) was classified as BGS since the kurtosis value of the corresponding segment was almost zero. These results show the performance of the mIKD algorithm used for selectively detecting inherent BS segments, despite the difference in the BGS level and in BSs amplitude and number.

Firstly, in order to determine the availability of the jitter and shimmer used in our algorithm, we compared the values of the selected features obtained from the normal subjects with those of the patients. As a result, both the jitters (*J_1, 3_*: 0.164 ± 0.007, *J_3, 3_*: 0.165 ± 0.006) and shimmers (*S_1, 2_*: 35.4 ± 1.7, *S_3, 2_*: 35.0 ± 1.7, *S_2, 2_*: 35.5 ± 1.0, *S_2, 1_*: 36.1 ± 1.7 dB) of the normal subjects were relatively higher than those of the patients (*J_1, 3_*: 0.143 ± 0.009, *J_3, 3_*: 0.151 ± 0.005, *S_1, 2_*: 31.2 ± 1.0, *S_3, 2_*: 30.9 ± 2.1, *S_2, 2_*: 31.8 ± 1.8, *S_2, 1_*: 31.2 ± 1.0 dB), whereas the CTTs of the normal subjects (20.4 ± 15.8 hours) were relatively lower than those of the patients (78.9 ± 14.0 hours) (*p *< 0.01).

**Figure 4:**
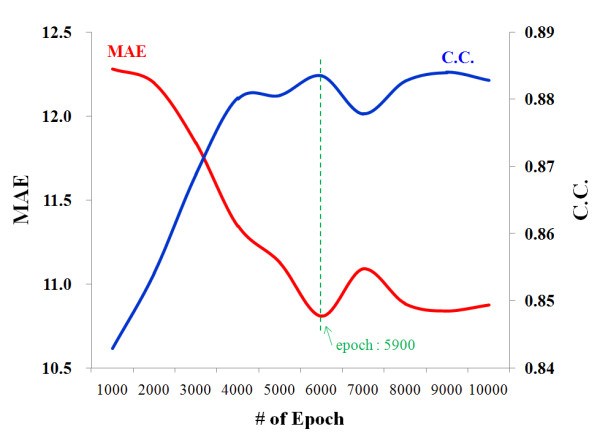
**Changes of the correlation coefficient (C.C.) and mean average error (MAE) of the testing-datasets according to the increase of the number of epoch**.

Next, for evaluating the performance of our algorithm, *k*-fold cross-validation (*k *= 3) was done using WEKA machine learning software (ver. 3.6. The University of Waikato, New Zealand) [22]. After the random rearrangement of all of 18 feature-datasets, 67% of them (12 feature-datasets) were used for training the BPNN model and the remaining 33% (6 feature-datasets) were used for estimating the CTT using the model trained previously. Consequently, the correlation coefficient, mean average error (MAE) and root mean square error (RMSE) between the measure CTTs and estimated values (eCTT) were 0.89, 10.6 and 14.6 hours at the number of epoch of 5,900, respectively, when applying the learning rate of 0.05 and the number of hidden nodes of 3 to the designed BPNN. Figure 4 shows the changes of the correlation coefficient and MAE of the testing-datasets according to the increase of the number of epoch.

## Discussion

Auscultation of the abdomen, one of physical examinations, has been used as a traditional technique for evaluating bowel motility. For the last few decades, there have been various comparative studies on the BSs of normal subjects and those of patients with bowel dysfunction, such as irritable bowel syndrome [5,6,8], Crohn's disease [6], diabetes mellitus [7] and obstruction [18], and their results showed significant differences between the features obtained from BS signals according to the pathological condition of bowel motility. Also, several researchers recently have developed the de-noising, segmentation and feature extraction strategies of BS signals based on various signal-processing techniques [9-16,19]. However, relatively few studies related to the quantitative estimation of bowel motility have been performed. Therefore, the goal of this study is to show the possibility of the quantitative estimation of the CTT, which has been used for the clinical assessment of bowel motility, by means of an ANN model and acoustical features.

BSs are produced from the movement of the intestinal contents, gas and fluid during peristalsis. Generally, normal 'very loud', 'gurgling' and 'rumbling' sounds are easily produced by a healthy bowel during an active stage of digestion. On the other hands, hyperactive BSs ('loud', 'high-pitched' and 'tinkling' sounds) might be caused by diarrhea or early intestinal obstruction, whereas hypoactive sounds (very diminished or absent sounds) are associated with bowel obstruction, paralytic ileus, torsion of the bowel or peritonitis that indicate diminished peristalsis [23,24]. As considering these differences of sounds, several informative BS-features related to the pathological condition of the gastrointestinal tract have been reported: time-domain features, such as sound-to-sound interval (silence between BSs durations) [5,6,14], number of BSs by time interval [4,6,11,14], sound index (sum of the BSs amplitude) [4,7], median duration [11,14,16] and median acoustic intensity [11,14] of BSs, and frequency-domain ones, such as main frequency of BSs [4,11,14,16,18].

Besides those features mentioned above, jitter and shimmer selected in our approach are measures of the fundamental frequency and amplitude cycle-to-cycle variations, respectively. They can represent the deviation or displacement of some aspect of the pitches of sounds in frequency- and time-domain, accordingly, they have been successfully used in a speaker verification, emotion expression, vocal or articular pathologies [20,25-27]. In this study, the jitters and shimmers were significantly different between two groups (*p *< 0.01) and highly correlated with the CTTs (correlation coefficient from -0.68 to -0.79). These differences were thought to be related to the delayed peristalsis caused by the impaired vagus and hypogastric nerves of the SCI patients [28], and this aspect might produce relatively decreased perturbation of the pitches of BSs. Therefore, the jitter and shimmer considered in our method could be employed as a useful clinical parameter for the continuous monitoring of the bowel motility.

In relation to recording-duration issues of our approach, the BS signals were analyzed based on short-term analysis that deals with small-duration samples of the entire activity in fasted humans, coincided with [5-8,18,19], and all of the features were obtained from impulsive BSs due to the management difficulty of subject's immobilization during the test and huge data. On the contrary, many researchers believe that the period of BS monitoring should be longer over at least 2 hours since lasting/regularly-sustained (RS) BSs monitored during a full migrating motor complex (MMC) cycle are also associated with bowel motility [4,10-16]. Unfortunately, even though the acoustical features obtained from the short-term recording of 10 minutes set in this study showed its feasibility, this strategy would raise a subject prolific of controversy related to the statistical reliability of the acquired samples. Therefore, in a future study we need to determine the appropriate recording period and to implement additional techniques for RS-BSs treatment as well as impulsive sounds.

Recently, the estimation method based on the regression analysis between the features obtained from BS signals, such as the jitter and shimmer, and conventional CTT was implemented in [17]. Accordingly, the comparison of the estimation results of the proposed approach by means of the ANN model with those obtained from the previous regression model was performed. When applying 18 feature-datasets used in this study into the regression model, the regression equation between the selected features and measured CTT was obtained as follows;(3)

Also, the correlation coefficient, MAE and RMSE between the CTTs and eCTTs were 0.89, 12.4 and 18.4 hours, respectively. As a result, the estimation errors of the ANN model (MAE of 10.6 and RMSE of 14.6 hours) were relatively lower than those of the regression model, whereas the correlation coefficients of both methods were almost same. Consequently, these results showed that the neural-network-based approach attempted in this study could enhance its performance.

The limitations of this study are the small number of subjects, and the effects of the physiological characteristics of the subjects, such as their body mass index or severity of the spinal cord impairment were not considered in our algorithm. In a future study, we will apply our algorithm to a larger number of patients with various bowel dysfunctions, as well as to normal subjects, in order to enhance the accuracy of the estimated CTT. Also, we plan to develop supplementary signal processing techniques for effectively reducing frictional noise generated unavoidably between the skin and microphone as well as unwanted bio-signals.

## Conclusions

A non-invasive algorithm for the estimation of the CTT based on BPNN model of the jitter and shimmer of the BS signals obtained by auscultation is reported. The correlation coefficient and MAE between the CTTs measured by the conventional method and the values estimated by our algorithm were 0.89 and 10.6 hours, respectively. The proposed algorithm showed good potential for the non-invasive measurement and continuous monitoring of bowel motility, instead of conventional radiography.

## List of Abbreviations

CTT: Colon Transit Time; MRI: Magnetic Resonance Imaging; BS: Bowel Sound; MLP: Multi-Layer Perceptron; ANN: Artificial Neural Network; IRB: Institutional Review Board; mIKD: Modified Iterative Kurtosis-based Detection; BGS: Back-Ground Sound; NF: Notch Filter; BPF: Band Pass Filter; BPNN: Back-Propagation Neural Network; eCTT: Estimated Colon Transit Time; MAE: mean average error; RMSE: root mean square error; MMC: Migrating Motor Complex.

## Competing interests

The authors declare that they have no competing interests.

## Authors' contributions

KK devised an estimation algorithm for bowel motility and implemented the portable system which consists of pre-processing circuits and data acquisition/analysis software using LabVIEW language. JS contributed to the experimental protocol for clinical assessment of bowel motility and interpretation of the results. CS supervised the project and contributed to the design of the algorithms. All authors read and approved the final manuscript.
